# Adopting the direct anterior approach: experience and learning curve in a Chinese patient population

**DOI:** 10.1186/s13018-019-1272-0

**Published:** 2019-07-16

**Authors:** Xiangpeng Kong, Luis Grau, Alvin Ong, Charlie Yang, Wei Chai

**Affiliations:** 1 Department of Orthopedics, Chinese PLA General Hospital, No.28 Fuxing Road, Haidian, Beijing, China; 2 The Rothman Institute, Thomas Jefferson University, Philadelphia, PA USA; 3 Colorado Joint Replacement, Porter Adventist Hospital, Denver, CO 80210 USA

**Keywords:** Direct anterior approach, Surgery complication, Learning curve, CUSUM

## Abstract

**Background:**

There are concerns regarding the complications encountered during the learning curve when switching to a direct anterior approach (DAA) for total hip arthroplasty (THA). The purpose of our study is to report our outcomes and complications after adopting a new approach in a Chinese patient population.

**Methods:**

From 2016 to 2018, a single surgeon’s first 100 cases with unilateral DAA for THA were reviewed. The patients were divided into 2 groups, the first 50 cases were designated as group A and the second 50 cases were designated as group B. The preoperative, intraoperative, and postoperative clinical data were analyzed. The cumulative summation method (CUSUM) was used to determine the learning curve.

**Results:**

There was a significant decrease in the complication rate from 44% in the first 50 cases to 16% in the second 50. The first 50 cases showed a significant increase in operating time, length of hospitalization, fluoroscopy, and complications. There was no significant difference in implant position, postoperative leg length discrepancy (LLD), Harris score, or creatine kinase. CUSUM analysis showed that complication rates and operating time reached acceptable and steady state after 88 cases and 72 cases respectively.

**Conclusions:**

Adopting DAA in a Chinese patient population has its own unique considerations and challenges. Even in the hands of an experienced surgeon, DAA is still a technically demanding procedure.

## Background

In recent years, the potential for rapid recovery after joint replacement surgery has attracted much attention in the field of arthroplasty [1]. It has been hotly debated if less invasive approaches are a potential tool in rapid recovery surgery [1–3]. Approaches that have been described as less invasive options for total hip arthroplasty (THA) include the mini-incision posterior, anterolateral, 2-incision, and the direct anterior approaches (DAA) [3]. The DAA, which was first described by Heuter, provides for a true intranervous and intermuscular exposure to the hip with preservation of the abductor musculature, external rotators, and posterior capsule [4]. As surgical techniques and instruments have improved for the DAA approach, several studies have shown advantages over other approaches, such as less soft tissue injury, reduced hospital stay, and a more rapid early recovery [5]. Despite these advantages, DAA’s demanding surgical technique and the potential need for auxiliary equipment have been reported as reasons which have limited further development [6, 7]. The aim of this study is to establish the learning curve of an experienced arthroplasty surgeon adopting DAA and to report the outcomes and complications throughout the learning curve in a Chinese patient population. We hypothesized that there is a higher complication rate and operative time early on in the learning curve. We also hypothesized that a steady state in terms of operative time and complications could be achieved.

## Patients and methods

Institutional Review Board approval for the study was obtained. A retrospective evaluation of the first 100 unilateral THA in 100 Chinese patients performed through DAA. The procedures were performed from January 2016 to January 2018 in a major academic center in China. Patients were excluded as candidates for DAA if body mass index (BMI) > 30 or severe hip dysplasia. The patients were divided into two groups. The first 50 cases (group A) and the second 50 cases (group B) (Table 1).

**Table 1:** Pre-operative data

Group	Age (years)	Gender (M/F)	BMI (kg/m^2^)	Diagnosis (ONFH ‘s proportion)	Pre-op Harris score
Group A	41.54 ± 4.08	32/18	21.79 ± 0.76	90.00%	51.09 ± 10.58
Group B	37.50 ± 3.86	30/20	23.37 ± 1.00	88.00%	54.09 ± 15.34
*t/λ2*	1.12	2.94	6.13	1.07	1.22
*p*	0.639	0.086	0.014	0.889	0.226

*M* male, *F* female, *BMI* body mass index, *ONFH* osteonecrosis of the femoral head

All cases were performed by a single surgeon. A tapered, cementless stem and cementless acetabular cup were used in all cases. The surgeon performed approximately 350 THA through a posterior lateral approach prior to converting to DAA. The surgeon did not perform DAA during any point of the residency or fellowship training. Prior to switching to DAA, the surgeon attended several cadaver courses and operative observations. The DAA surgical technique which previously published by one of the senior authors in this study was used for all patients [8].

The patients were followed for 3 months to identify complications and functional outcomes. Operative time, length of hospitalization (LOH), post-operative leg length discrepancy (post-op LLD), post-operative Harris score, perioperative complications, fluoroscopic time, and creatine kinase levels (before surgery and the third day after surgery) were documented for every patient. Surgical complications were defined as entering the incorrect interval during the approach, periprosthetic fracture, significant vascular injury (vascular surgeon intervention required), incision-related complications, dislocation, prosthetic joint infection, heterotopic ossification, implant malposition, unacceptable LLD, and reoperation.

Anteroposterior pelvic X-ray of patients was obtained 3 months postoperatively. The radiographs were assessed for fractures, prosthetic loosening, presence of heterotopic ossification (Brooker Stage), leg length discrepancy, and acetabular and femoral component position. The measurements were performed on digital radiographs using the measurement software package by Orthoview Systems. Leg lengths discrepancy (LLD) was measured by drawing a line across the base of the acetabular teardrops and referencing that line to a fixed point on the lesser trochanter, the LLD > 10 mm was recorded as unacceptable [9]. The femoral stem position was measured by drawing an angle between prostheses’ longitudinal axis and femoral anatomical axis, the angle > 3° was recorded as unacceptable [10]. The acetabular cup abduction angle was measured by drawing an angle between the cup long axis and the acetabular teardrops, the abduction angle > 50° or < 30°was recorded as unacceptable [11]. (Tables 2 and 3).

**Table 2:** Post-operative Data

Group	Operating time (min)	Post-op LLD (mm)	LOH (days)	Post-op Harris score	Fluoroscopic time	Creatine kinase (D3–0) (U/L)
GroupA	113.44 ± 30.13	5.04 ± 3.10	6.60 ± 2.95	83.19 ± 7.53	4.80 ± 1.40	936.44 ± 478.01
GroupB	86.66 ± 21.45	3.74 ± 2.36	5.40 ± 2.12	84.22 ± 5.66	2.86 ± 1.01	654.62 ± 443.99
*t/λ2*	5.12	2.26	2.34	1.03	8.81	0.03
*p*	0.000	0.136	0.021	0.906	0.004	0.866

Explanation: *post-op* post-operative, *LLD* leg length discrepancy, *LOH* length of hospitalization; *D3–0* the difference of creatine kinase between the third day after surgery and before surgery

**Table 3:** Complications

Group	Intraoperative Complication				Unacceptable LLD (> 10 mm)	Malposition of stem	Malposition of cup	Incision-related complication	Dislocation	HO	Total
	Total	1	2	3							
Group A	5	1	3	1	4	2	3	4	2	2	22
Group B	2	2	0	0	2	2	1	1	0	0	8
*t/λ2*	0.61				0.17	0.00	0.26	0.84	0.49	0.49	9.33
*p*	0.433				0.674	1.000	0.610	0.469	0.496	0.495	0.002

Explanation: *1* wrong interval; *2* periprosthetic fractures; *3* major vascular injury. *LLD* leg length discrepancy, *HO* heterotopic ossification

A learning curve is defined as an improvement in performance over time or with increasing experience or training. The cumulative summation method (CUSUM) is a sequential analysis tool that was initially used in industrial settings for quality control purposes. It can be used to establish the learning curve for a surgical procedure and allows one to judge when an individual’s performance has achieved a predefined level of competence [12, 13]. For CUSUM analysis, four parameters are defined: the acceptable failure rate (p0), the unacceptable failure rate (p1), the type I error rate (α), and the type II error rate (β). The equations shown in Table 1 are used to calculate the CUSUM score.

We reviewed lots of literature to determine the category and incidence of complication of DAA. According to two studies of high quality and large sample size, we defined the acceptable DAA minor and major complication rate as 5% and the unacceptable complication rate as 20% [7, 14]. Our study’s protocol, including the criterion of inclusion and exclusion, surgical technique, and the definition of implication, were similar to the reference literature. So we defined the acceptable DAA minor and major complication rate as 5% and the unacceptable complication rate as 20%. The probabilities of α and β were set at 0.05 and 0.20, respectively. The results of CUSUM analysis are presented in a chart with case numbers plotted on the *x*-axis and the corresponding CUSUM score on the *y*-axis. This allows performance over consecutive procedures to be visualized. When a failure occurred, the constant ‘1-S’ was added to the cumulative score. When a success occurred, the variable ‘s’ was subtracted from the cumulative score. Hence, success is rewarded by a downward slope whereas failure is represented by an upward slope on the chart. If the line crosses the upper decision limit (h1) from below, this indicates that the actual failure rate is equal to the unacceptable failure rate with a type I error. If the line crosses the lower decision limit (h0) from above, this indicates that the actual failure rate does not differ from the acceptable failure rate with a type II error probability of 0.20. When the line is between h1 and h0, no statistical inference can be made [13].

## Results

There were 62 males and 38 females included in our study. Preoperative diagnoses included osteonecrosis of the femoral head (89 cases), developmental dysplasia of the hip (6 cases), primary osteoarthritis (2 cases), rheumatoid arthritis (2 cases), and one case of pigmented villonodular synovitis. There were no significant differences between the two groups in demographic data. (Table 1).

There was a significant decrease in overall complication rate from 44% in the first 50 cases to 16% in the second 50 cases. The first 50 cases showed an increase in operating time, fluoroscopic time, LOH, and incidence of complications that were statistically significant. There was no significant difference between the two groups in blood transfusion rate, implant position, postoperative LLD, postoperative Harris score, or creatine kinase level. There were no infection, aseptic loosening, and reoperation in two groups. Table 2 summarizes our intraoperative and postoperative results.

**Fig. 1:**
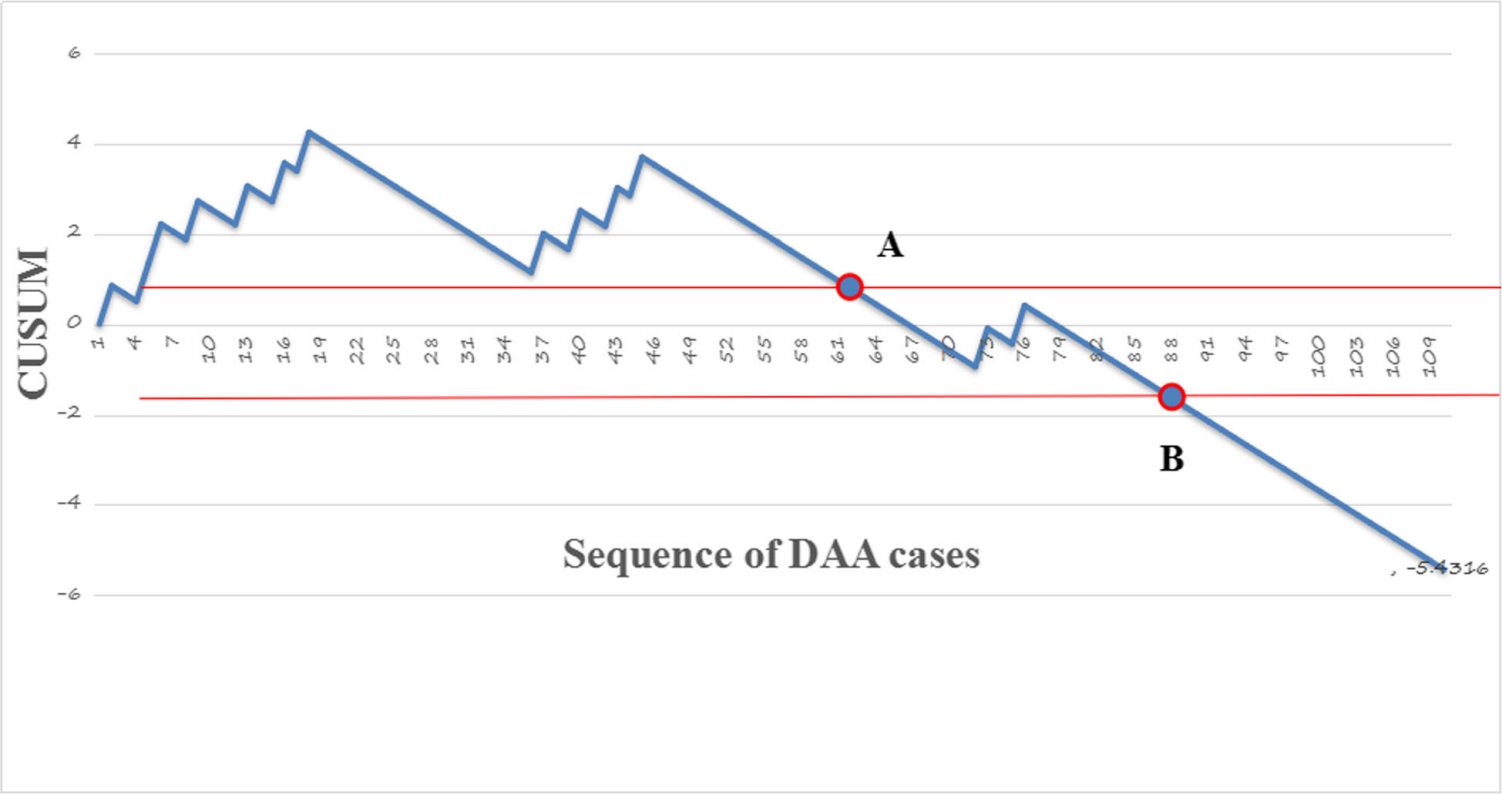
The CUSUM learning curve. The CUSUM learning curve shows that after 88 cases an acceptable major and minor complication rate was achieved

**Fig. 2:**
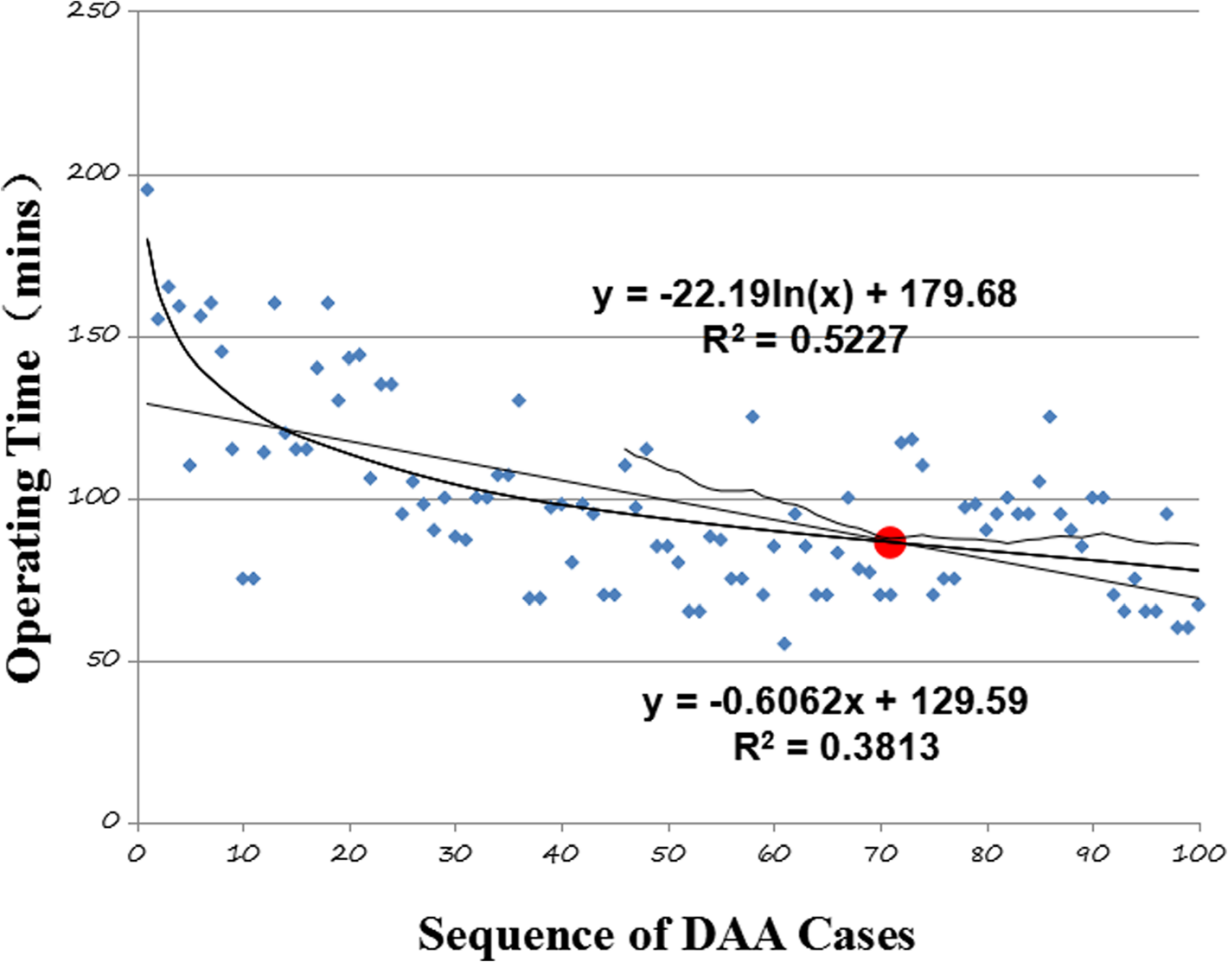
The CUSUM learning curve of operating time. The tendency chart and linear fitting equation of operating time shows that steady state was reached by the 72nd case

The CUSUM learning curve chart (Fig. 1) shows that 62th case corresponds to the main inflection point (point A) at which the failure rate became consistent. The 88th case (point B), the line crosses the lower decision limit and the failure rate is equal to the defined acceptable failure rate (H0, 5%). The failure rate before the 62th case remained the unacceptable threshold (H1, 20%). The tendency chart and linear fitting equation of operating time (Fig. 2) shows that the approximate 72th case reached a steady state. After 72 cases, operating time normalized.

## Discussion

Preservation of the abductor muscle, posterior capsule, and external rotators, the potential to minimize leg length discrepancy, lower dislocation rates, and a faster postoperative recovery are attractive reasons for considering the direct anterior approach to THA [15]. However, these benefits do not come without a well-reported increased complication rate early in the learning curve, even in the hands of experienced surgeons [16–20].

Our complication rates in a Chinese population are similar to other DAA learning curve studies published in the literature. Woolson reported a 9% incidence of major complications in a group of community orthopedic surgeons in their learning curve with the direct anterior approach [14]. The overall complication rate in our study of 30% seems high compared to their findings; however, Woolson’s definition of complication was less stringent than ours. In the current study, we included errors in the surgical approach, implant malposition, periprosthetic fractures, wound complications, dislocations, and HO as complications. By the criteria used in Woolson’s study, our complication rate was similar at 6%. Using our criteria for complications, we found an increase in completion rates in our first 50 cases as compared to our second 50 cases which were statistically significant.

Several authors have concluded that femoral exposure is the most difficult aspect of the DAA, with an increased risk for iatrogenic femoral fractures. Our femoral fracture rate with the DAA of 3.0% was similar to other respective series [19, 21–23]. Similarly, Lee found a 2.3% intraoperative fracture rate [23]. It is important to note that our femoral fracture rate in our second 50 cases was 0%. Masonis [19] similarly noted in his series that after 62 cases no intraoperative calcar fractures occurred. We attribute our improvement throughout the learning to improved soft tissue dissection technique and exposure of the femur as well as to gaining experience for the feel of broaching the femur through the DAA. We recommend surgeons take their time when learning this technique especially on femoral exposure. Proceeding to prepare the femur without adequate exposure can lead to fractures.

One of the benefits of the DAA is the ability to use intraoperative fluoroscopy to assist with implant positioning and leg length. In agreement with the results published by Masonis [19], we found that with the routine use of intraoperative fluoroscopy in our DAA cases, accurate leg length within 10 mm was achieved in 94% of cases. The incidence in patient-perceived leg length discrepancies declined from 8% in the first 50 cases to 4% in the second 50 cases. We noted that in the first 50 cases, we tended to make the operative leg longer, which is similar to the results published by Woolson [14]. We attribute these findings to our tendency to sacrifice leg length equality for additional stability when we used the posterior approach. As we became convinced of the inherent stability of the DA approach, we felt more comfortable striving for even leg lengths even in hips which did not feel as tight as we were used to putting in when using a posterior approach. We recommend surgeons utilize the benefit of fluoroscopy and supine positioning to decrease the likelihood of complications from LLD.

Lewinnek described the acetabular safe zone with regards to the dislocation between 30° and 50° of acetabular abduction [24]. Acetabular component abduction angle measurements demonstrated that only 4% of all DAA cases were beyond the safe zone. Seventy-five percent of the cases outside of the safe zone occurred in the first 50 patients. This is similar to the 93% of cups in the safe zone using fluoroscopy with a DAA reported by Suarez [25]. Masonis also noted excellent acetabular cup position with the routine use of fluoroscopy with the DAA [19]. In terms of femoral component malposition, 4% of all femoral components were put into varus or valgus > 3°.

We attribute our improved accuracy for component position to feeling more comfortable making adjustments to implant position based on fluoroscopic images as we gained more experience. We believe that accurate implant position and preservation of the external rotators and posterior capsule was in part responsible for our low 2% dislocation rate in our first 100 cases. Our results are comparable to those of Masonis who also reports a 2% dislocation rate in the learning curve [19]. As our exposure technique improved and we learned to used fluoroscopy to guide our implant position, our dislocation rate dropped to 0% in our last 50 cases.

Muscle damage can be measured by serum creatine kinase level [15]. In our study, although the postoperative creatine kinase’s differences were not significantly different, we did notice a trend towards higher serum levels in the first 50 cases. This may suggest that decreased muscle damage through lower surgical time and improved dissection technique may be part of the reason why we noted a significant decrease in hospital stay in our second 50 cases as compared to our first 50. However, larger studies are likely needed to detect a statistically significant difference. The heterotopic ossification rate, which some believe is related to soft tissue damage was stable at 2% over the course of the learning curve. We attribute these low numbers to irrigating all cases with 3 L of normal saline before closing. We noticed that our incidence of HO decreased after we routinely irrigated and became more mindful of injury to the tensor muscle with a retractor.

Our results show a statistically significant decrease in the use of intraoperative fluoroscopy after our first 50 cases. The approximately 5 s and 3 s of fluoroscopic time in our first 50 and second 50 cases respectively were similar to those reported by Pomeroy [26]. At this rate, a surgeon would need to perform more than 300,000 DAA total hips to be at increased risk for cataracts. In our experience, we noted that early on we used significantly more fluoroscopy to help guide us through the surgery. Moreover, we noted that our struggles with exposure led to us relying on fluoroscopy to reassure ourselves that the components were in an acceptable position. This is especially true since the anatomic landmarks are very different in a supine position through a DAA as compared to a standard posterior lateral approach. Masonis also noted a significant decrease in the use of fluoroscopy between their first 50 cases and second 50 cases of their learning curve [19]. However, their fluoroscopy time was significantly higher than ours at 32.1 and 14.5 in their first 50 and second 50 cases, respectively.

The CUSUM curve is acknowledged as one of the most effective and accurate methods to define a learning curve for a procedure. CUSUM analysis showed that a minor and major complication rate of 5% was achieved after 88 cases. Moreover, it took 72 cases for surgical time normalize at just under 90 min. De Steiger reported that 400 cases were required to achieve a surgical time similar to a posterior lateral approach in surgeons adopting the DAA [17]. While they reported a longer learning curve for surgical time to reach a steady state, our surgical time at the end of our learning curve was under 90 min which was similar to theirs.

In most studies, the DAA learning curve mainly focused on operating time and complications [15–20]. Our study is unique in that we also reported on implant position, leg length discrepancy, functional outcomes, and markers for muscle damage. Moreover, our study is the first to describe the DAA learning curve in a Chinese patient population which has unique characteristics. Only 6% of patients underwent THA for primary osteoarthritis in our study. Nearly 90% of the patients in our study underwent THA for osteonecrosis. This is not uncommon in a Chinese patient population where reported rates of primary OA are much lower than in North American patients. Moreover, at an average BMI of just over 20, our patient population was much smaller than that of the North American surgeons [15, 26].

Although surgeons operating on a thinner patient population without the contracture and deformity associated with end-stage OA may expect to have a less difficult time adopting the DAA, the small working space and canal diameter in a smaller Chinese patient population coupled with limited resources in the OR in many parts of China and the tendency for patients to allow disease processes to progress due to limited access to healthcare, can present unique challenges when adopting the DAA. We can conclude from our study that even in the hands of an experienced surgeon in a major university hospital and with carefully selected patients who were thin and without severe deformity, there is a significant learning curve with a relatively high complication rate associated with adopting the DAA approach in a Chinese patient population.

There are several factors listed above which we attribute our improvement in complication rate and outcomes. Another important factor in decreasing complications in the learning curve is continued education. After 20 cases of DAA surgery, the senior surgeon again participated in cadaver courses and did a visitation with an experienced DAA surgeon. We suggest that surgeons adopting the DAA attend cadaver course and visitation prior to their first case. Then after several cases return for a second course and visitation to learn tips on the parts of the case they may be having trouble with.

Our study does have some limitations. By virtue of the study design, the result of a single experienced surgeon may not be reproducible for all surgeons. A larger sample may have shown statistically significant differences in clinical outcome and specific complications between cases early and late in the learning curve. For example, a study with a larger sample size is likely needed to detect a difference in Harris hip scores or to detect a significant difference in creatine kinase levels.

## Conclusions

Adopting the DA approach in a Chinese patient population has its own unique considerations and challenges. To our knowledge, this is the only study describing the DAA learning curve in a Chinese patient population. Moreover, it is the only learning curve study for DA approach reporting on the learning curve for surgical time and complications as well as on functional outcomes, implant position, leg length discrepancy and markers for muscle damage. The results of our study show that although DAA has the potential for improved implant position, lower dislocation rates, shorter hospital stay, and a more rapid early recovery as compared to other approaches, it is a technically demanding procedure that is associated with a high complication rate early in the learning curve, even in the hands of an experienced surgeon in carefully selected cases. Eighty-eight cases are required before complication rates normalize and 72 cases before surgical times normalize. Even in the hand of a high volume surgeon performing more than 300 total joint arthroplasties per year, it still required well over 1 year to achieve proficiency with the DAA. We therefore recommend that low volume surgeons think carefully if the potential benefits of the DAA outweigh the complication risk imparted on their patients during their learning curve. Surgeons who decide to take on the challenge of the learning curve should attend courses and visitations to familiarize themselves with the critical steps to the approach, instrumentation prior to performing their first cases.

## Data Availability

All data generated or analyzed during this study are included in this published article.

## Abbreviations

BMI: Body mass index
CUSUM: Cumulative summation method
DAA: Direct anterior approach
HA: Heterotopic ossification
LLD: Leg length discrepancy
LOH: Length of hospitalization
OA: Osteoarthritis
ONFH: Osteonecrosis of the femoral head
THA: Total hip arthroplasty
